# The symbol of spread of modern Western botany into China: *Chih-wu hsüeh*, an unconventional translation in the late Qing dynasty

**DOI:** 10.1007/s13238-017-0452-0

**Published:** 2017-07-28

**Authors:** He Zhang

**Affiliations:** 1 Department for the History of Science and Scientific Archaeology, University of Science and Technology of China, Hefei, 230026 China; 2 School of Marxism, Bengbu Medical College, Bengbu, 233030 China

Traditional botany in ancient China mainly included agriculture and medicine. In contrast, botany in the broader modern sense was introduced from the West and established in China gradually. In 1858, the London Missionary Society Press (Mo Hai Shu Kuan) in Shanghai published a translation titled *Chih-wu hsüeh* (*Botany*) (Fig. 1), which was compiled and translated by Alexander Williamson and Joseph Edkins, two British missionaries (Fig. 2), and Shanlan Li (Fig. 3), an exceptional scholar in the late Qing dynasty. As the first translated work on modern Western botany, this translation signified the spread of modern Western botany into China. Therefore, it served as the enlightenment in the history of modern Chinese botany and was regarded as a historic event in the history of the eastward spread of Western learning. The publication of *Chih-wu hsüeh* was an example of the successful scientific and cultural interaction between Western and Chinese ideas because it has been acknowledged as the fusion of traditional Chinese botany and modern Western botany.

**Figure 1:**
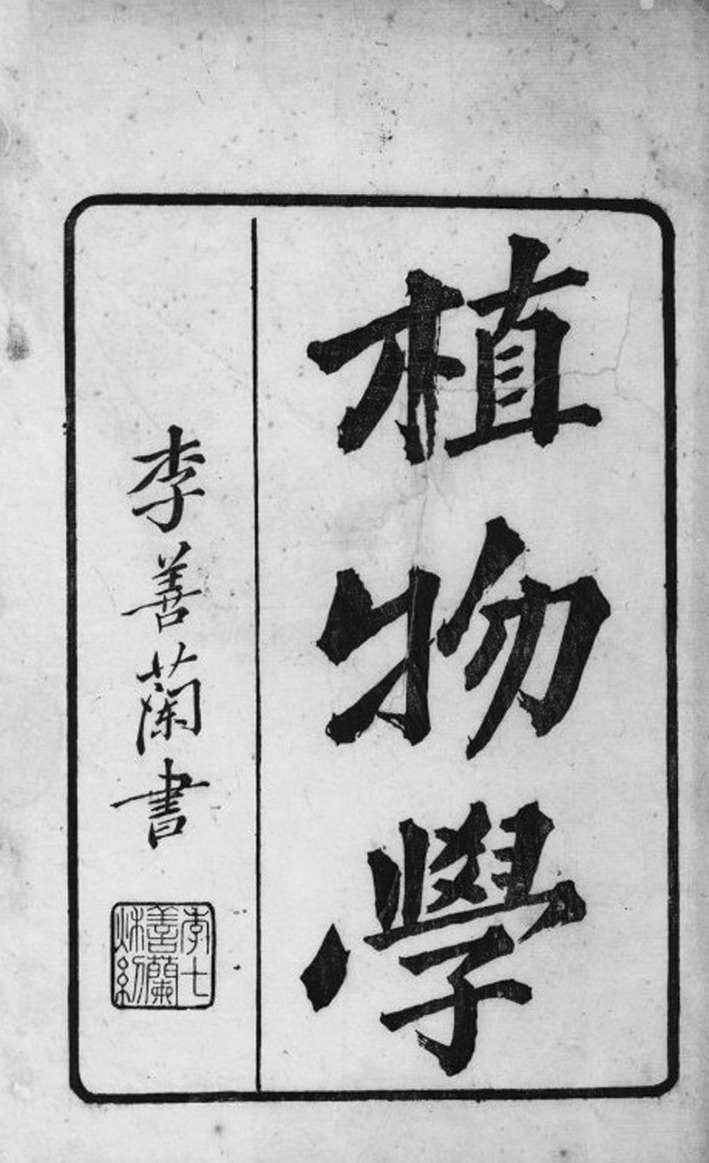
*Chih-wu hsüeh*, by Alexander Williamson, Joseph Edkins, and Shanlan Li in 1858

**Figure 2:**
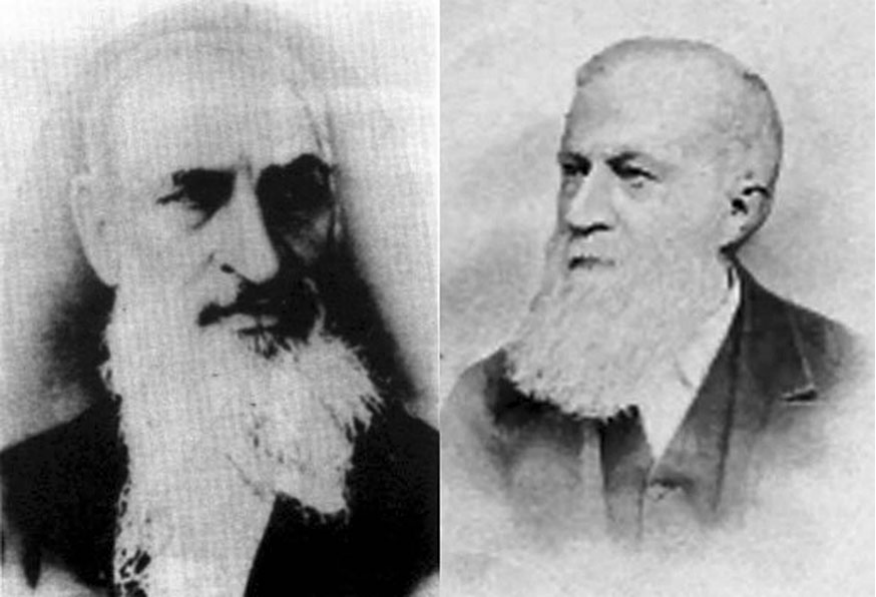
Alexander Williamson (1829–1890) and Joseph Edkins (1823–1905)

**Figure 3:**
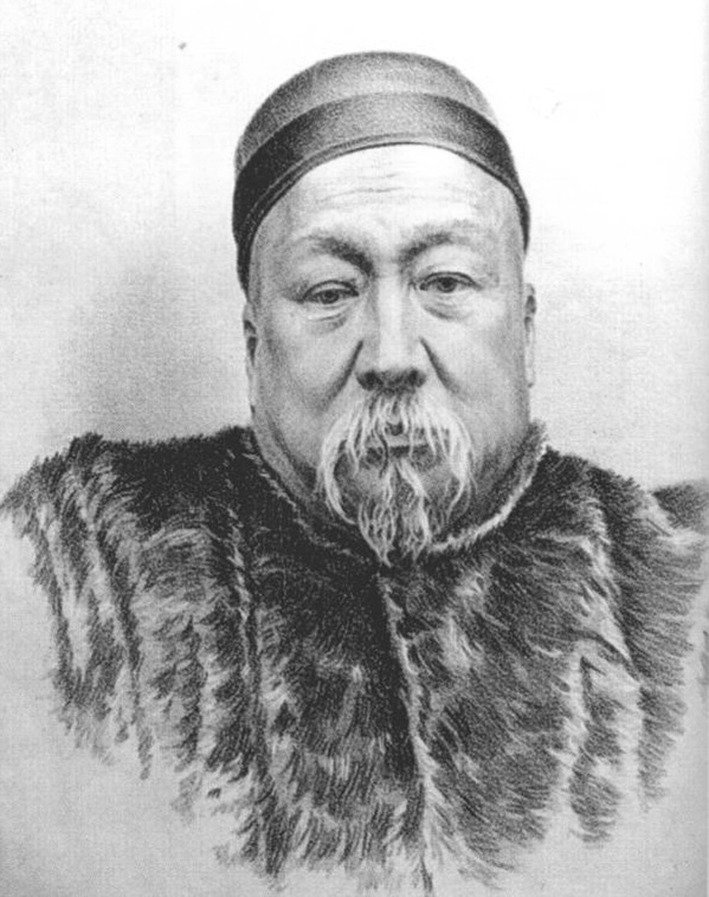
Shanlan Li (1811–1882)

For a long period of time, the original foreign source of *Chih-wu hsüeh* had generally been regarded as *Elements of Botany* by British botanist John Lindley. However, the year, edition, and place of publication of the source text were all unknown. Later, it was claimed that the original source was the fourth edition of *The Outline of the First Principles of Botany* written by Lindley and published in London in 1841 (Pan, 1984). However, following studies questioned this claim, and the issue had remained unresolved for a long time. Recently, studies concluded that there were four original sources of *Chih-wu hsüeh*, including Lindley’s 1847 or 1849 *The Elements of Botany* (Fig. 4) (Shen, 2000), John H. Balfour’s 1851 *Phyto-Theology* (Fig. 5) (Lu, 2015), Lindley’s 1853 *The Vegetable Kingdom* (Fig. 6), and Volume 1 of *Chambers’s Information for the People* (Fig. 7) published by William and Robert Chambers in 1848.^1^ Although it was not rare for a contemporary translation of a science book to have more than one original source, none had as many as *Chih-wu hsüeh* with four different sources.

**Figure 4:**
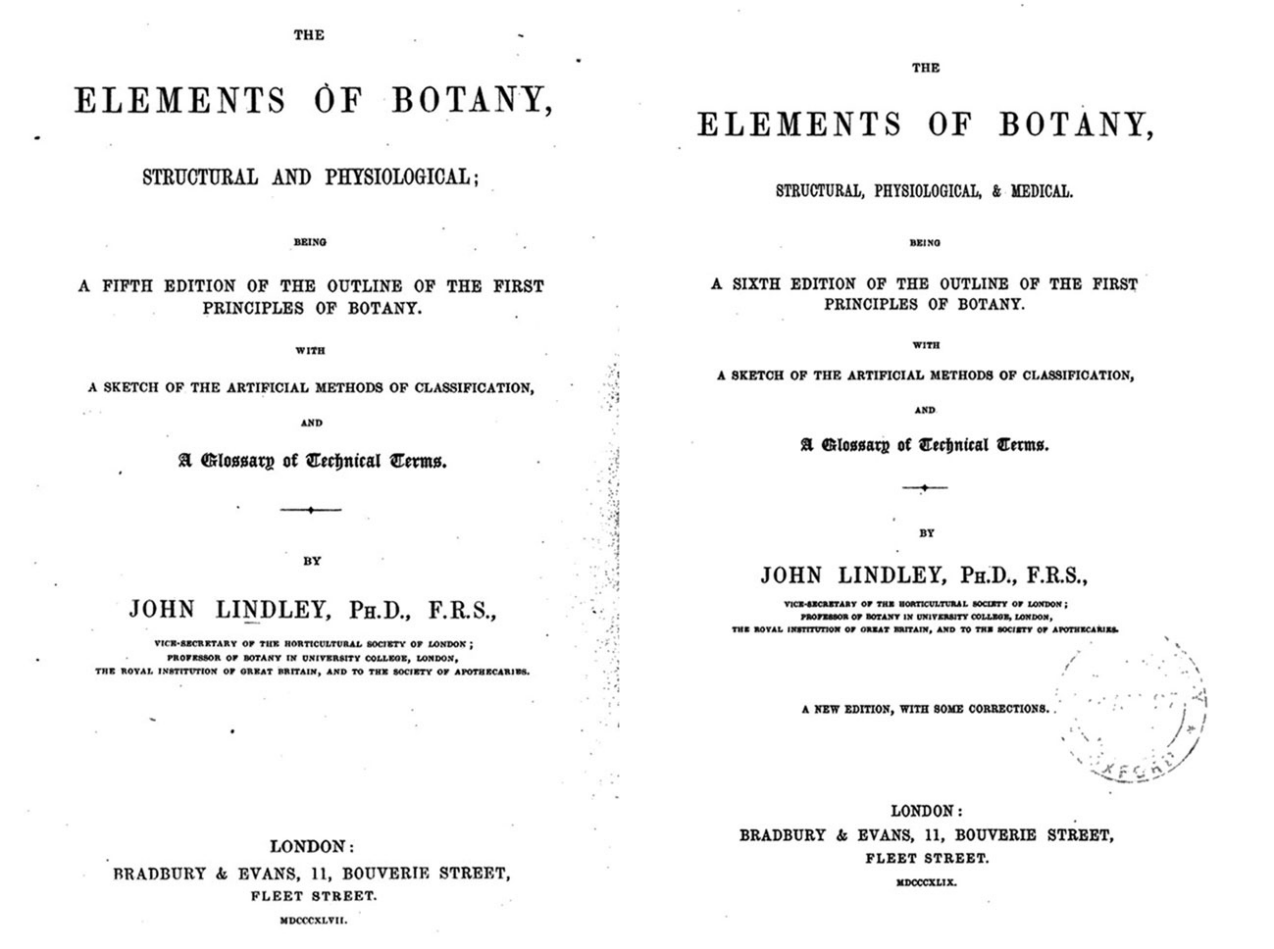
*The Elements of Botany*, by John Lindley in 1847 and 1849

**Figure 5:**
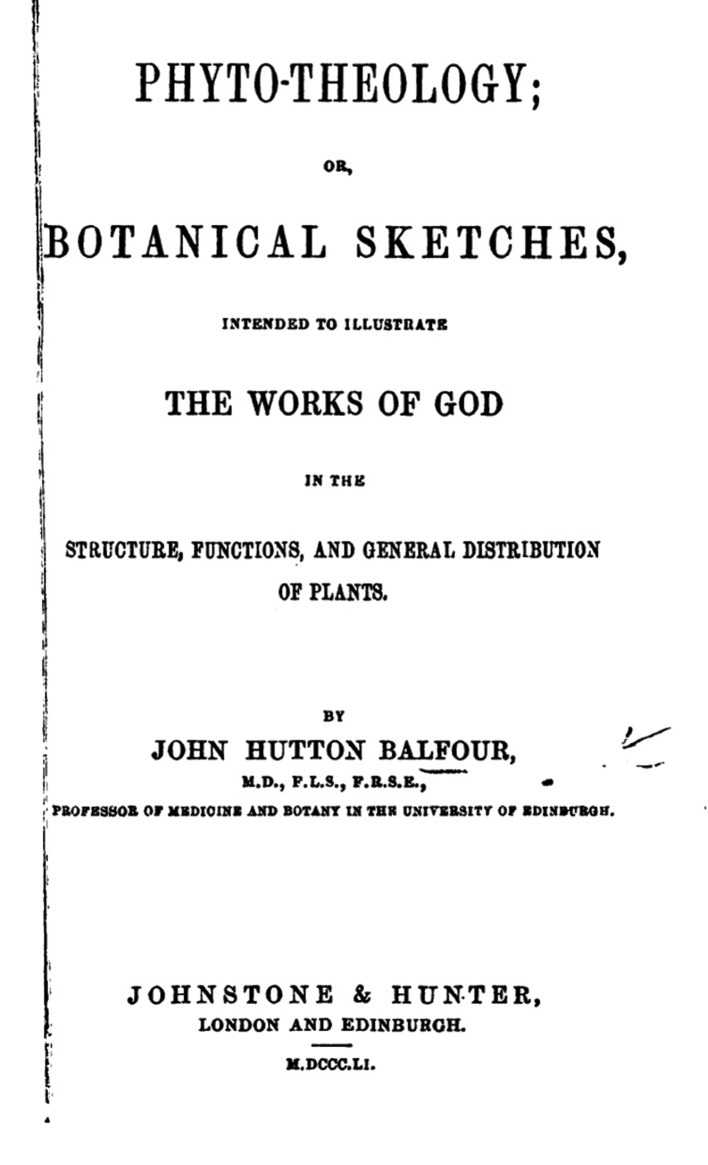
*Phyto-Theology*, by John H. Balfour in 1851

**Figure 6:**
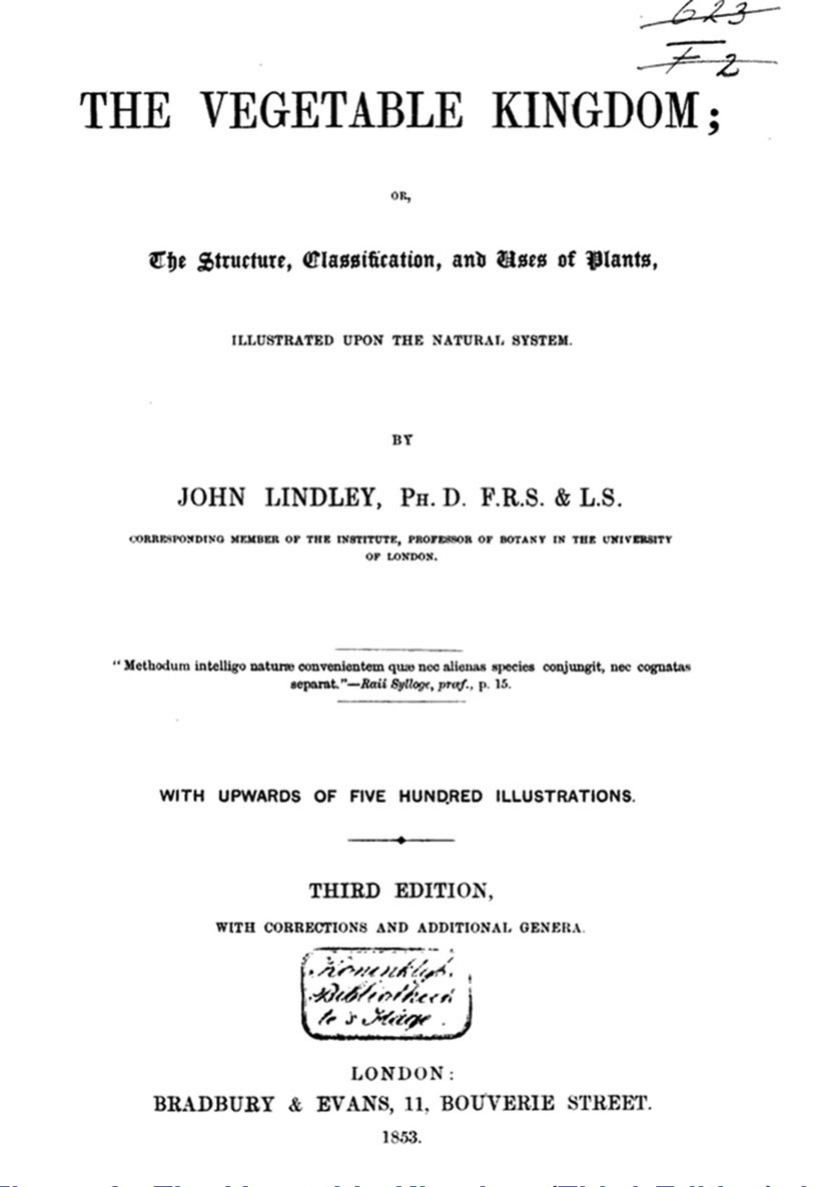
*The Vegetable Kingdom* (Third Edition), by John Lindley in 1853

**Figure 7:**
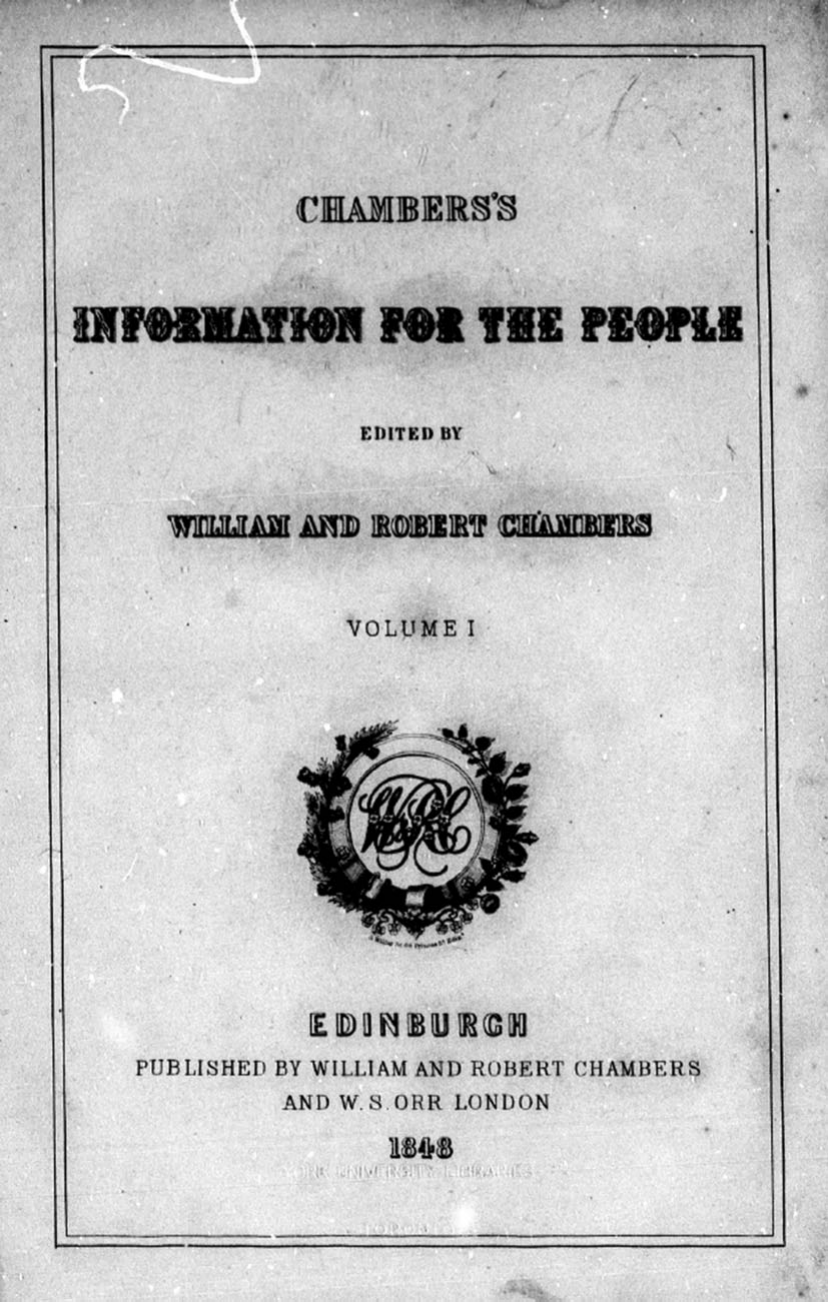
Volume 1 of *Chambers’s Information for the People*, by William and Robert Chambers in 1848

*Chih-wu hsüeh* consisted of eight volumes with about 35,000 Chinese characters and more than 200 illustrations. In the preface, Li provided the background, main content, and significance of this work. The first volume was a general discussion of the extensive use of plants and the significance of botany, the fact that all animals and plants are composed of cells, the similarities and differences between plants and animals, and the geographical distribution of plants. The main content of *Chih-wu hsüeh* began in the second volume and could be divided into two major parts: part one was morphology, structure, and physiology of plants; part two was plant taxonomy. Part one could be further divided into “lun nei-ti” (on the inner body) and “lun wai-ti” (on the exterior body). The second volume introduced the inner structure of plants, describing the four kinds of plant tissues termed in Chinese “ju bao ti” (聚胞体), “mu ti” (木体), “xian ti” (线体), and “ru lu ti” (乳路体), which corresponded to cellular tissue, woody tissue, vascular tissue, and laticiferous tissue in the original text, respectively. The third to sixth volumes described the morphological structures and physiological functions of the roots, stems, branches, leaves, flowers, fruits, and seeds, as well as recently acquired knowledge on transpiration, photosynthesis, and fertilization of plants. Part two was included in the last two volumes, which integrated the seven classes of natural system for arranging plants established by Lindley (1853) into five “lei” (类): “wai zhang” (外长), “nei zhang” (内长), “shang zhang” (上长), “tong zhang” (通长), and “ji sheng” (寄生). Every “lei” could be further divided at levels of “bu” (部), “xiao-bu” (小部), “ke” (科), “zu” (族), and “zhong” (种). The classification criteria were described, and 37 “ke” among the total 303 “ke” classified were described in detail. In addition, the seventh volume also introduced “cha-li zhi fa” (察理之法) which documented the observational and experimental methods of botany, including a small section on the preparation and identification of herbarium samples. Moreover, it was worth noting that the book also included considerable content devoted to natural theology (Liu, 2008).

*Chih-wu hsüeh* was completed by the three translators in the London Missionary Society Press, founded by the British missionary Walter Henry Medhurst in 1843. The press was a noted missionary organization engaged in translation work and was one of the publishing centers of missionary translations. Williamson and Edkins were both British missionaries who came to China to promote religious propaganda in 1855 and 1848, respectively. They joined the press to translate English books. They did science translations in the belief that science would be an “auxiliary to the spreading of the gospel” (Wright, 1998). Li was born in Haining, Zhejiang Province and had been obsessed with mathematics since childhood. To understand the development of modern Western mathematics, he also joined the press in 1852 and participated in the translation of Western science texts into Chinese. With different motivations, the three translators met each other at the press and began a highly effective cooperation. Williamson and Edkins had scientific literacy and, in particular, the former had expertise on botany. Since Li could not read English and Williamson and Edkins had only a limited knowledge of Chinese language and culture, they adopted a translation method termed in Chinese “xi yi zhong shu” (西译中述), meaning interpretation by the Westerner and transcription by the Chinese. Specifically, the foreign collaborator interpreted the original text into colloquial Chinese, and then Li transcribed the oral translations into literary Chinese manuscripts. Whenever difficulties were encountered, the collaborators needed to communicate with each other repeatedly, and sometimes, comprehension of meaning required facial expressions and body language, such as gestures by the foreigner. After translation was accomplished, Li would read and revise the entire manuscript, and the translated texts would then be elaborated and polished to be made as simple and readable as possible for the Chinese people. “This demanded a thorough understanding of the scientific issues as well as a high level of literary skill (Wright, 1998).”

According to the preface by Li, the first seven volumes of *Chih-wu hsüeh* were translated by Williamson and himself, and the last volume was translated by Edkins and himself after Williamson returned home due to illness. Although Li did not understand English, he not only transcribed and polished the manuscripts but also, importantly, translated botanical names and terms. Li made supreme efforts to avoid transliteration of the sounds into Chinese characters. In contrast, he used the existing traditional Chinese botanical names and terms and thereby linked the corresponding concepts between Chinese and Western botany, making the translation accessible. For example, he used the character “xu” (须) for stamen and “xin” (心) for pistil. For new concepts that did not have equivalents in traditional Chinese botany, he coined neologisms, some of which are still in use today. For example, botany was translated as “chih-wu hsüeh”, cell was translated as “xi bao” (细胞), and order was translated as “ke”^2^.

“The communication between the East and the West is more difficult in that there are not only language barriers but also divergent cultural patterns (Tsien, 1954).” While translating modern Western botany theory, Li endeavored to combine it with traditional Chinese botanical knowledge and integrate it into the Chinese cultural background in collaboration with his foreign partners. For instance, when describing the changes during the process of seed germination, Li explained the transformation from albumen to sugar using the example from people in Jiangnan of China. They used germinated wheat to produce sugar and wine. This example made the Western theory more acceptable to the Chinese people. Therefore, *Chih-wu hsüeh* was certainly not a simple passive translation of original texts, but a demand-oriented innovative work that assimilated Western knowledge and culture into a form accessible to the Chinese people. In this sense, it was a creation rather than a translation.

A few years after its publication, *Chih-wu hsüeh* was introduced to Japan and played an active role in the popularization of modern botanical knowledge and the evolution of botanical nomenclature in Japan. It generated a significant influence on the development of modern botany in Japan, which had a profound impact on China as a result of the indirect importation of Western learning from Japan at the beginning of the 20th century.

## Footnotes

1 The last two original sources were identified from the author’s textual research.

2 Currently, order is translated as “mu”, and “ke” is the translation of family. At that time, family was not a taxonomic category.
